# Impact of COVID-19 on acute appendicitis presentation, management and pathology findings in adult and paediatric populations

**DOI:** 10.1371/journal.pone.0300357

**Published:** 2024-04-17

**Authors:** Dorothy B. Johnston, Helen G. Coleman, David Colvin, Suzanne Lawther, Maurice B. Loughrey

**Affiliations:** 1 Centre for Public Health, Queen’s University Belfast, Belfast, United Kingdom; 2 Department of Paediatric Surgery, Royal Belfast Hospital for Sick Children, Belfast Health and Social Care Trust, Belfast, United Kingdom; 3 Department of Cellular Pathology, Royal Victoria Hospital, Belfast Health and Social Care Trust, Belfast, United Kingdom; Acibadem Maslak Hospital: Acibadem Maslak Hastanesi, TURKEY

## Abstract

**Background:**

We investigated the impact of the COVID-19 pandemic on trends of presentation, management and pathology findings in patients who underwent an appendicectomy for suspected acute appendicitis.

**Method:**

The retrospective study reviewed patients (n = 939 adults and n = 329 children) who had an appendicectomy performed for suspected acute appendicitis and histopathology assessment in the Belfast Health and Social Care Trust, Northern Ireland. Pre-COVID-19 (March 2019 to February 2020) and COVID-19 Year 1 (March 2020 to February 2021) data were compared. Chi-squared tests were applied to compare timeframes.

**Results:**

513 adult appendicectomies were performed in the immediate year pre-COVID-19, compared to 426 in COVID-19 Year 1, representing a 17% reduction. No such reduction was seen within the paediatric population, likely related to a change in regional paediatric referral criteria during the pandemic. When comparing COVID-19 Year 1 with pre-pandemic, fewer patients presented with <24 hours of symptoms (45% v 53%, p = 0.005), and there was greater use of pre-operative computed tomography imaging in adults (63.2% v 48.7%, p<0.001). Fewer adult and paediatric cases of simple acute appendicitis and non-diagnostic specimens, with relative increased proportions of perforated acute appendicitis, were observed in COVID-19 Year 1 compared with pre-pandemic. No absolute increase in perforated acute appendicitis cases was observed in adults.

**Conclusion:**

Year 1 of the COVID-19 pandemic was associated with delayed presentation of acute appendicitis in adults and children. In adults, an overall reduction in appendicectomy operations, increased use of pre-operative diagnostic imaging, and fewer specimens showing simple acute appendicitis or non-diagnostic features, collectively support appropriate restriction of surgery for those patients with a more certain acute appendicitis diagnosis.

## Introduction

Acute appendicitis (AA) is one of the most common causes of acute abdominal pain and is the most common acute surgical emergency worldwide [1,2]. A systematic review by Ferris *et al*. suggested the pooled incidence of appendicectomy or appendicitis was 151 per 100,000 person-years in western Europe [3]. Diagnosis is usually based either on a clear clinical diagnosis or via imaging modalities including ultrasound scan (US), computed tomography (CT) or, in specific circumstances, magnetic resonance imaging (MRI) [4,5]. The treatment of choice for AA is surgical, in the form of either laparoscopic or open appendicectomy. In cases where the diagnosis is clinically suspected but not definitive, often a diagnostic laparoscopy is first performed, followed by appendicectomy if the diagnosis is confirmed. Laparoscopic appendicectomy is currently viewed as the gold standard treatment in adults with AA [6,7]. Appendicectomy specimens are routinely sent to histopathology for analysis, a diagnostically important step, firstly, to confirm a diagnosis of AA, which may only be evident microscopically, and, secondly, to exclude underlying pathology, especially neoplasia, which may be incidental or the cause of the AA [8,9].

When the SARS-CoV-2 (COVID-19) pandemic first began to impact the United Kingdom (UK) in March 2020, many non-essential operations were cancelled, due to initial safety fears surrounding COVID-19 [10,11]. In late March 2020, the four main surgical colleges in the UK and Ireland as well as the Association of Surgeons of Great Britain and Ireland (ASGBI), Association of Coloproctology of Great Britain and Ireland (ACPGBI) and Association of Upper Gastrointestinal Surgeons (AUGIS) issued intercollegiate general surgery guidance [12]. In relation to the management of suspected AA, this guidance advised that patients should have non-operative management where feasible, avoidance of laparoscopy unless necessary, due to the risks of aerosol generation, and CT imaging of the chest in any patient undergoing a CT scan for diagnosis [12]. By early June 2020, guidance was updated to begin to cautiously reintroduce laparoscopy again where certain strict safety criteria were met [13].

The aim of this study was to investigate the impact of the COVID-19 pandemic, and associated intercollegiate guidance, on trends of clinical presentation, surgical management and pathology findings of patients who underwent an appendicectomy for suspected AA, over a two year time period.

## Methods

The retrospective observational descriptive study design included patients of all ages who underwent an appendicectomy operation between 1^st^ March 2019 and 28^th^ February 2021. To allow analysis of the impact of COVID-19 on clinical and pathology trends of interest, data were divided into a pre-COVID-19 pandemic timeframe (March 2019-February 2020 inclusive), and a COVID-19 pandemic Year 1 timeframe (March 2020-February 2021 inclusive), based on the experience of COVID-19 in the UK. Study cases comprised all appendicectomy specimens submitted for histopathology assessment during this time identified from the laboratory information management system of a large pathology department. This provides pathology services to a region covering two of the five Health and Social Care Trusts in Northern Ireland, as well as the regional paediatric service delivered by the Royal Belfast Hospital for Sick Children (RBHSC). As of mid-2020, the populations served by the Belfast, and South-Eastern, Health and Social Care Trusts totalled approximately 723,421 individuals, representing 38% of the 1.9 million Northern Ireland population [14]. Importantly, during the primary surge of COVID-19 in Northern Ireland in early 2019, working practices for children with acute surgical conditions changed. Some children with suspected AA were referred to RBHSC, whereas previously most such care was provided by adult surgical services in district general hospitals. As the study design was a retrospective clinicopathological service evaluation, ethical approval was not required.

Of the initial specimens identified (n = 1,677), elective or incidental appendicectomies, or appendicectomies performed as part of another surgical operation, for example resection of proximal colonic or ovarian cancer, were excluded. This left an analytical dataset including 1,268 patients with appendicectomy performed either as an emergency for a presumed clinical diagnosis of AA, or performed at a later date (up until end of case note review date of January 2022) as an interval appendicectomy, after previous conservative management.

Patient hospital electronic healthcare records, including appendicectomy pathology reports, were reviewed by a surgical registrar (DBJ). Data collected included patient date of birth, sex, symptom duration prior to admission, if first presentation to hospital, if interval appendicectomy, if failed conservative management on this or previous admission, pre-operative imaging use, surgical technique (laparoscopic/open/converted), primary pathology findings, any additional pathology findings and COVID-19 infection status. Admission date, surgery date and discharge date enabled calculation of age at surgery, time from admission to surgery and length of stay. Imaging recorded the most superior imaging modality utilised for that patient [15,16] e.g., if they had US and then CT, only CT was recorded. Given the retrospective clinical casenote review nature of the study, some data items were unavailable for some patients and were recorded as “unknown”.

All appendicectomy specimens were subject to macroscopic and microscopic pathology assessment as part of routine specimen handling and processing and reported by one of a team of consultant gastrointestinal pathologists. No review of pathology specimens was conducted for this study. Findings from descriptive pathology reports were classified as: normal, simple AA (without perforation), AA with perforation, chronic inflammation (no acute changes evident) or secondary inflammation (serosal-based inflammation without evidence of transmural inflammation to indicate primary AA). Simple AA included all severities of inflammation lacking perforation, with perforation considered the most reliable pathological indicator of complicated AA in a study based on free text reports from multiple pathologists. In addition to the primary pathology assessment of the inflammatory state of the appendix, other additional pathology findings, both neoplastic and non-neoplastic, were recorded separately. These may have been incidental or causative with respect to the AA. Any ambiguous pathology reports lacking clarity regarding pathology diagnosis were reviewed by the study pathologist (MBL) to determine appropriate classification.

### Statistical analysis

Data were compiled within a Microsoft Excel data extraction table. All statistical analysis was carried out using STATA version 16.2 (StataCorp, TX, USA). Analyses compared findings in the pre-COVID-19 pandemic timeframe (March 2019-February 2020) to the COVID-19 pandemic Year 1 timeframe (March 2020-February 2021). Additional analyses restricted comparisons to April-June 2020 with April-June 2019, representing the first three months of the COVID-19 pandemic after guidance was issued from UK surgical colleges [12]. Chi-squared tests were applied to estimate differences between study periods for the overall patient group, and for adults and children separately. Adults were defined as patients aged 18 years or older at the date of admission.

## Results

**Fig 1:**
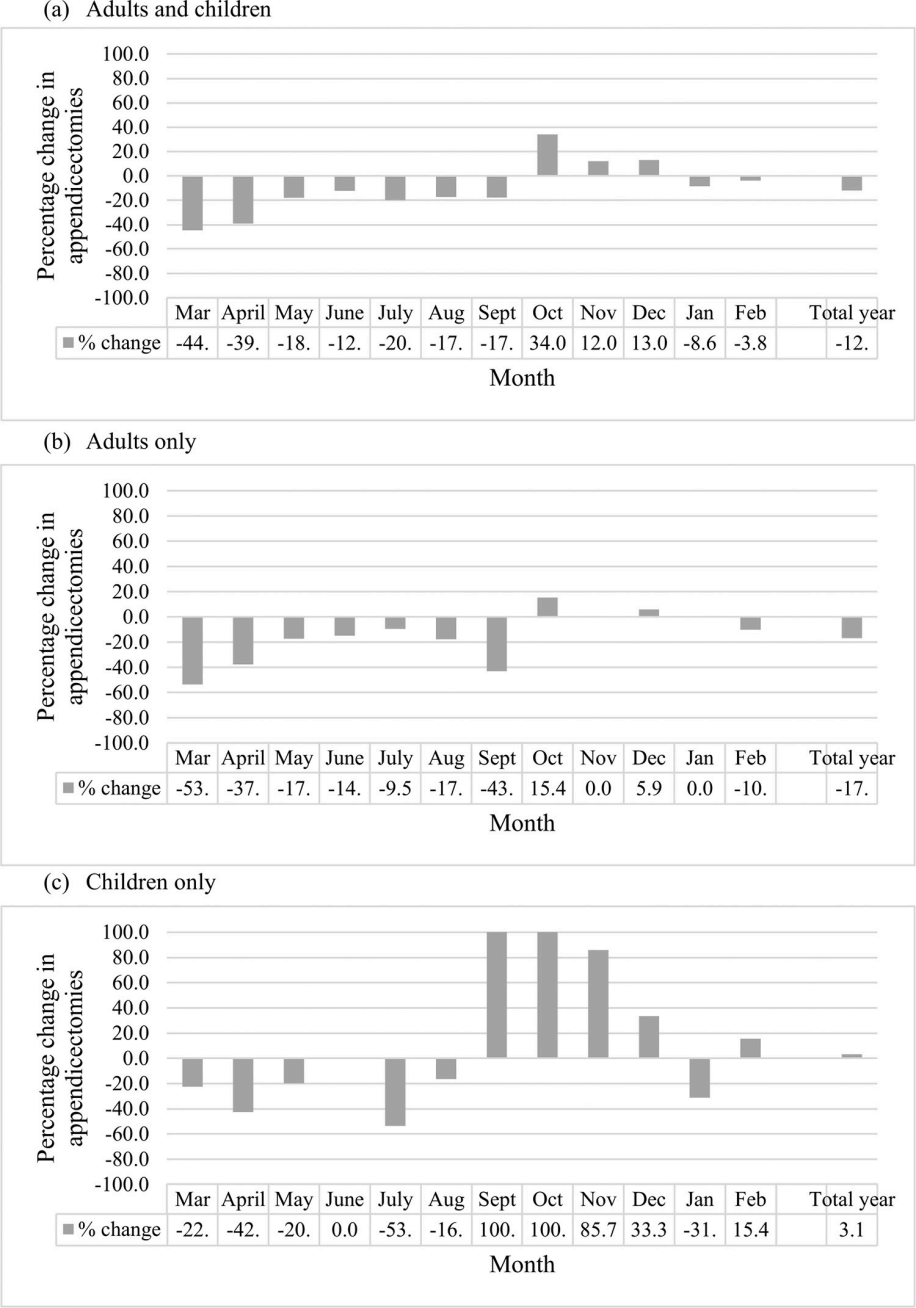
Percentage change in numbers of appendicectomies performed in the (a) overall (adults and children), (b) adults only, and (c) children only patient groups comparing pre-COVID-19 timeframe to the COVID-19 pandemic Year 1 timeframe, month by month.

Of the 1,268 patients (n = 939 adults and n = 329 children) identified to have an appendicectomy for suspected AA during the two year study time period, 675 presented within the pre-COVID-19 timeframe and 593 within the COVID-19 pandemic Year 1 timeframe. This represented an overall 12.1% reduction in appendicectomies performed during the first COVID-19 year compared to pre-COVID-19. This difference was only apparent in adults, representing a 17% reduction, with no decrease in appendicectomies performed in the paediatric patients between pre-pandemic and Year 1 pandemic timeframes. However, when examining monthly data, there was a marked reduction in appendicectomies in both adults and children in the first six months, compared to corresponding pre-pandemic monthly data (Fig 1). This was compensated, within the paediatric population only, by a marked increase in the second six months.

The age at date of surgery for patients with suspected AA ranged from 1–90 years overall. The median age of adults was 35 years (interquartile range 26–48 years) and the median age of children was 11 years (interquartile range 8–15 years). 58% of patients were male. Age and sex profiles were similar for both age groups and did not differ between COVID-19 pre-pandemic and pandemic Year 1 timeframes. Only four patients tested positive for SARS-CoV-2 on or during admission.

Tables 1 and 2 summarise the clinical, surgical and pathological findings, comparing the pre-COVID-19 pandemic and pandemic Year 1 timeframes. Data is presented for all patients and separately for adult and paediatric age groups.

**Table 1:** Clinical and surgical findings related to appendicectomy procedures.

	All			Adults			Children		
	Pre-COVID-19 pandemic n = 675 (%)	COVID-19 pandemic Year 1 n = 593 (%)	*p* value	Pre-COVID-19 pandemic n = 513 (%)	COVID-19 pandemic Year 1 n = 426 (%)	*p* value	Pre-COVID-19 pandemic n = 162 (%)	COVID-19 pandemic Year 1 n = 167 (%)	*p* value
Symptom duration prior to admission									
≤24 hours >24hours Interval Unknown	330 (53.2) 290 (46.8) 11 44	245 (45.0) 300 (55.1) 9 39	0.005*	250 (53.5) 217 (46.5) 9 37	183 (46.5) 211 (53.6) 5 27	0.038*	80 (52.3) 73 (47.7) 2 7	62 (41.1) 89 (58.9) 4 12	0.050*
Imaging US CT MRI Nil	98 (14.5) 253 (37.5) 0 (0.0) 324 (48.0)	64 (10.8) 278 (46.9) 4 (0.7) 247 (41.7)	0.001	52 (10.1) 250 (48.7) 0 (0.00) 211 (41.1)	24 (5.6) 269 (63.2) 3 (0.7) 130 (30.5)	<0.001	46 (28.4) 3 (1.9) 0 (0.0) 113 (69.8)	40 (24.0) 9 (5.4) 1 (0.6) 117 (70.1)	0.220
Time to surgery									
≤24 hours 25–48 hours >48 hours	595 (88.2) 63 (9.3) 17 (2.5)	522 (88.0) 55 (9.3) 16 (2.7)	0.980	444 (86.6) 55 (10.7) 14 (2.7)	368 (86.4) 44 (10.3) 14 (3.3)	0.871	151 (93.2) 8 (4.9) 3 (1.9)	154 (92.2) 11 (6.6) 2 (1.2)	0.731
Surgical Technique									
Laparoscopic Open Converted Unknown	436 (64.6) 199 (29.5) 36 (5.3) 4 (0.6)	369 (62.2) 195 (32.9) 27 (4.6) 2 (0.3)	0.517	335 (65.3) 140 (27.3) 34 (6.6) 4 (0.8)	279 (65.5) 121 (28.4) 25 (5.9) 1 (0.2)	0.656	101 (62.4) 59 (36.4) 2 (1.2) 0 (0.00)	90 (53.9) 74 (44.3) 2 (1.2) 1 (0.6)	0.355
Length of stay									
≤48hours 49-72hours >72hours	355 (52.6) 114 (16.9) 206 (30.6)	318 (53.6) 92 (15.5) 183 (30.8)	0.801	278 (54.2) 91 (17.7) 144 (28.1)	232 (54.5) 75 (17.6) 119 (27.9)	0.997	77 (47.5%) 23 (14.2%) 62 (38.3%)	86 (51.5) 17 (10.2) 64 (38.3)	0.508

Percentages may not total 100% due to rounding.

*p value compares symptom duration prior to admission, excluding interval and unknown cases.

US, ultrasound scan; CT, computed tomography; MRI, magnetic resonance imaging.

**Table 2:** Pathology findings related to appendicectomy procedures.

	All			Adults			Children		
	Pre-COVID-19 pandemic n = 675 (%)	COVID-19 pandemic Year 1 n = 593 (%)	*P* value	Pre-COVID-19 pandemic n = 513 (%)	COVID-19 pandemic Year 1 n = 426 (%)	*P* value	Pre-COVID-19 pandemic n = 162 (%)	COVID-19 pandemic Year 1 n = 167 (%)	*P* value
Normal Simple AA Perforated AA Chronic Secondary inflammation	77 (11.4) 393 (58.3) 159 (23.6) 32 (4.7) 14 (2.1)	49 (8.3) 337 (56.9) 183 (30.9) 18 (3.0) 6 (1.0)	0.007	57 (11.1) 291 (56.8) 124 (24.2) 28 (5.5) 13 (2.5)	31 (7.3) 250 (58.6) 128 (30.0) 12 (2.8) 5 (1.2)	0.012	20 (12.4) 102 (63.0) 35 (21.6) 4 (2.5) 1 (0.6)	18 (10.8) 87 (52.1) 55 (32.9) 6 (3.6) 1 (0.6)	0.194

Percentages may not total 100% due to rounding. AA: acute appendicitis.

### Clinical presentation

There was some evidence of delayed presentation with symptoms during the COVID-19 pandemic Year 1, with 45.0% of patients presenting within <24 hours of symptom onset compared with 53.2% in the pre-COVID-19 pandemic timeframe (p = 0.005). This difference was evident within both adult and paediatric populations. Numbers of interval appendicectomy procedures were small, representing <2% of all patients in both timeframes (Table 1).

### Diagnostic imaging

**Fig 2:**
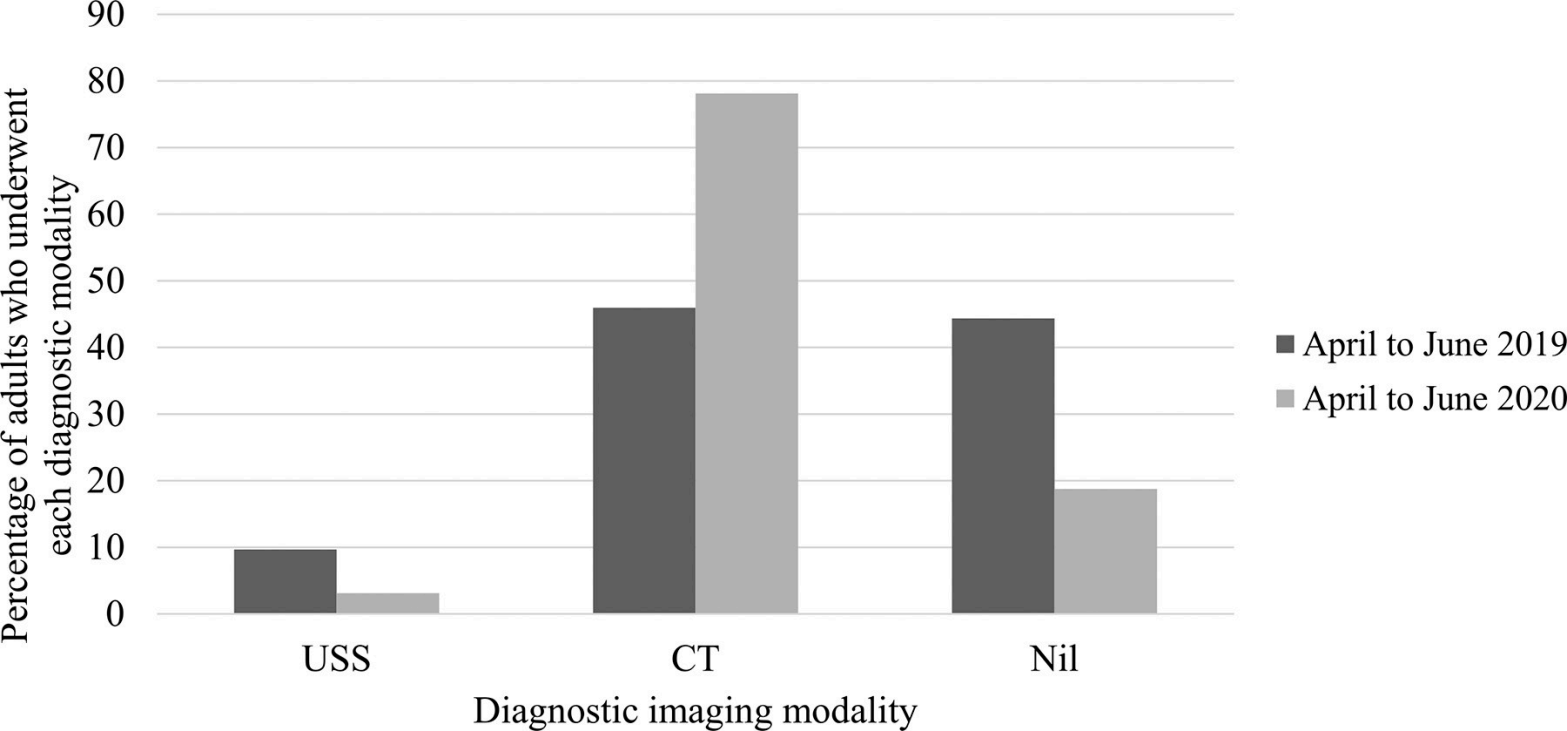
Percentage changes in diagnostic imaging modalities applied to adults presenting with suspected acute appendicitis comparing pre-COVID-19 to COVID-19 pandemic April to June timeframes. US, ultrasound scan; CT, computed tomography.

Adults were much more likely to undergo imaging for suspected AA than children (Table 1). There was notably greater use of pre-operative CT imaging during the COVID-19 Year 1 timeframe compared with pre-pandemic in adults (63.2% v 48.7%, p<0.001). In analysis comparing April-June 2020 with April-June 2019, this difference in pre-operative CT scanning was even more pronounced in adults (78.1% v 46.0%, p<0.001), depicted in Fig 2. More frequent pre-operative CT imaging did not apply to the paediatric population, most of whom had no imaging or US only, with minimal reliance on CT imaging (Table 1). No differences in imaging modalities in children were observed when comparing pre-pandemic and COVID-19 pandemic Year 1 timeframes (p = 0.22). MRI scanning was utilised in only a very small number of patients.

### Patient management

Greater use of pre-operative imaging did not translate into any significant delays in time to surgery between the two study timeframes, with 88% of patients with suspected AA receiving their surgery within 24 hours of presentation (Table 1).

**Fig 3:**
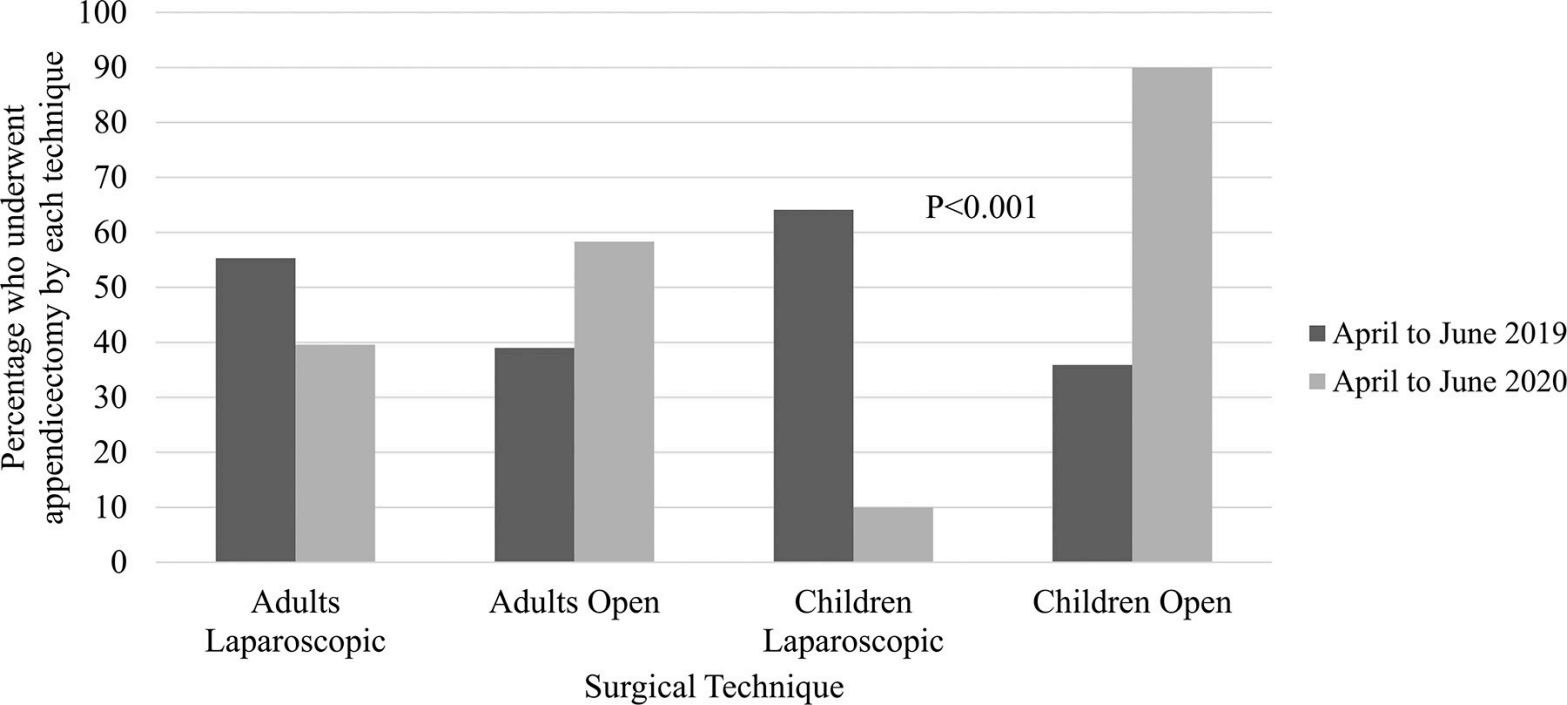
Surgical technique adopted for appendicectomies, for adults and children separately, comparing pre-COVID-19 to COVID-19 pandemic April to June timeframes.

Regarding surgical technique, approximately two thirds of patients underwent laparoscopic surgery and there were no significant differences between the pre-pandemic and COVID-19 pandemic Year 1 timeframes (p = 0.517, Table 1). However, when restricting comparisons to the three month timeframes, only 39.6% of adult appendicectomies were performed laparoscopically in April-June 2020, compared to 55.3% in April-June 2019 (p = 0.013). This difference was even more marked within the paediatric population, only 10% of whom underwent a laparoscopic appendicectomy, compared to 64.1% pre-COVID-19 (p<0.001, Fig 3). The observed increased frequency of open surgery did not translate into longer lengths of hospital stays (Table 1).

### Pathology findings

**Fig 4:**
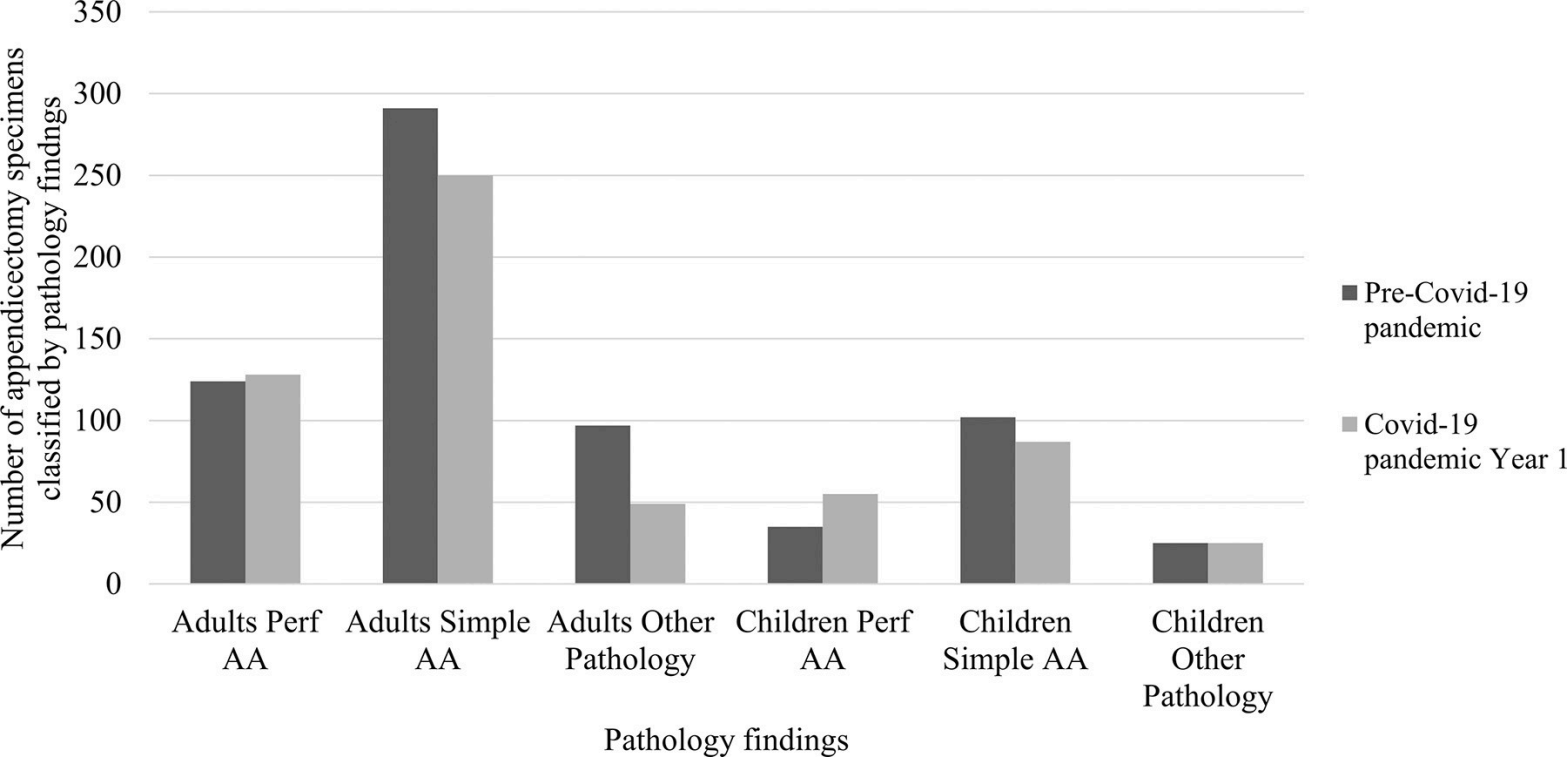
Number of appendicectomy specimens classified by pathology findings, comparing adults and children separately pre-COVID-19 timeframe to the COVID-19 pandemic Year 1 timeframe. “Other pathology” comprises normal, secondary or chronic inflammation without changes of primary AA. AA, acute appendicitis; Perf, perforated.

There was a significant relative increase in diagnoses of perforated AA in the COVID-19 Year 1 pandemic timeframe, compared with pre-pandemic (30.9% v 23.6%, p = 0.007, Table 2, Fig 4). However, absolute numbers of adults with perforated AA remained relatively stable, whilst fewer adult cases displayed simple AA, chronic or secondary inflammatory changes only, or were histologically normal (p = 0.012). In contrast, there was an increased proportion and absolute number of specimens demonstrating perforated AA within the paediatric population (32.9% v 21.6%, p = 0.021, Table 2, Fig 4), and an approximately commensurate reduction in cases diagnosed with simple AA. Fewer paediatric appendicectomy specimens were diagnosed pathologically as normal or displaying chronic or secondary inflammatory changes only, compared to the pre-pandemic timeframe, although this difference was non-significant (p = 0.194).

There was no significant difference in additional pathology findings reported, with similar numbers of non-neoplastic and neoplastic findings evident in the pre-COVID-19 pandemic and COVID-19 pandemic Year 1 timeframes (Table 3).

**Table 3:** Additional pathology findings in appendicectomy specimens.

	Pre-COVID-19 pandemic	COVID-19 pandemic Year 1
Diverticular disease	34	28
Pinworms	8	5
Endometriosis/ endosalpingiosis	4	5
Actinomyces infection	1	1
Granulomas	3	3
Neoplasms*	11	12

These findings may have been incidental or, for some, predisposed the patient to acute appendicitis.

*Neuroendocrine tumour, goblet cell adenocarcinoma, mucinous neoplasm, serrated polyp or leiomyoma.

## Discussion

This retrospective observational descriptive study included adults and children and compared patients who underwent appendicectomy for presumed AA during the 12 months after the onset of the COVID-19 pandemic with patients from the preceding year. The main findings were a reduction in overall numbers of operations during the pandemic timeframe, notably within the first six month period, evidence of some delay in clinical presentation, within adults and children, and greater use of diagnostic CT imaging within the adult group. Much less laparoscopic surgery was employed during the early months, especially in children. Pathology data indicated significantly fewer adult appendicectomy specimens showing simple AA or non-diagnostic features, with an accompanying increase in relative, but not absolute, numbers of specimens demonstrating perforated AA.

The impact of the pandemic on the incidence and management of AA has been extensively studied and reported, both in adults and in children. Systematic reviews have reported a clear increase in adoption of non-operative pathways and in complicated or perforated AA within the paediatric population during the pandemic [17–22]. However, the situation is less clear regarding changes in overall incidence of AA in children and adults and regarding management and outcomes of AA in adults, with systematic review and meta-analysis which includes both adult, paediatric and mixed populations highlighting conflicting results [21].

Regarding incidence, we found a 17% reduction in cases for adults during the 12 month study period of the pandemic, which was comparable to other studies [21,23,24]. A systematic review and meta-analysis by Kohler *et al*. found a 20.9% reduction in number of adult cases of appendicitis/appendectomies during the COVID-19 pandemic, compared with similar pre-pandemic timeframes [21]. This study included two very large, German, population-based studies which reported reductions of cases of 12.9% and 18.9%, respectively [25,26]. Explanation for this reduction is likely multifactorial, including but not restricted to: reluctance of patients to present to hospital because of a fear in contracting COVID-19, more plausible with milder symptoms, and encouragement by authorities to avoid unnecessary hospital presentations. This interpretation is supported by our finding of significantly fewer patients presenting during the pandemic with less than 24 hours duration of symptoms, which has also been previously reported [21].

There was also a significant reduction in paediatric appendicectomies during the pandemic timeframe, but this was restricted to the first six month period of analysis. This likely reflects greater adoption of a non-operative management (NOM) pathway in the early months of the pandemic [18,22]. For example, Emile *et al*. found by meta-analysis of fourteen studies that application of NOM during the COVID-19 pandemic was significantly more likely than before the pandemic (OR = 6.7, p < 0.001) [18]. One UK-based, multicentre study reported that 39% of paediatric cases of AA during the first two months of the COVID-19 pandemic followed a NOM pathway [27]. NOM was not directly assessed by the current study, as cases were identified by pathology specimen rather than clinical presentation, but greater application of NOM has been previously reported for the paediatric population within the same early pandemic time period [28]. That no reduction in paediatric appendicectomies was evident comparing the full twelve month time periods likely reflects the wider paediatric catchment area resulting from a change in referral practice during the pandemic, rather than any change in management policy. The change in regional policy leading to a wider catchment area resulted in a marked increase in paediatric appendicectomy numbers in the second six months of the study period, compared to prepandemic, entirely compensating for reduced numbers in the first six months of the pandemic. No such pattern was evident for the adult population comparing the same time periods, consistent with a stable adult study population.

During Year 1 of the COVID-19 pandemic study period, we observed more frequent use of pre-operative CT imaging in the adult population, particularly in the three months following issuance of clinical guidance in this respect [12]. There was no such uptake of CT imaging within the paediatric population, likely due to greater concerns about radiation exposure [29] and the potential need for sedation or an anaesthetic in some younger children prior to CT [30]. As with the pre-pandemic period, clinical examination and laboratory testing remained the diagnostic mainstay for children. In adults, the increased use of pre-operative imaging is consistent with the practice of encouraging greater diagnostic certainty prior to operating and restriction of surgery for those most in need, in keeping with overall patterns of reduced elective and emergent surgical activity during the pandemic [31,32]. This also likely contributed to the reduction in observed adult case numbers during the pandemic period.

Following publication of intercollegiate surgical guidelines regarding avoidance of laparoscopic surgery [12], there was a predictable marked increase in open appendicectomy procedures, particularly within the paediatric population. Once this guidance was eased, resumption of normal laparoscopic activity resumed and no differences were apparent at 12 months, in adult or paediatric populations. These findings are consistent with those of other studies which reported on this aspect of management [21,33].

The pathology findings between time periods enhance overall clinical interpretation of the data presented. The adult population demonstrated a reduction in absolute numbers of diagnoses of AA during the pandemic, along with reductions in negative (normal) appendices and in those demonstrating features of chronic appendicitis or secondary (serosal) appendiceal inflammation but lacking histological features diagnostic of primary AA. These findings likely reflect greater use of imaging in adults, resulting in greater diagnostic certainty pre-operatively and consequently fewer operations performed on patients who did not have AA. Importantly, the observed reduction in overall AA incidence was not accompanied by any increase in absolute incidence of perforated AA in adults. This adds support to those studies with similar findings, and the conclusion that AA may be a non-progressive disease and a subset may be safely treated conservatively with antibiotics [34–36]. For example, Neufeld *et al*. reported a 29% reduction in adults presenting with AA during the COVID-19 pandemic, without any associated increase in the incidence of complicated AA [23]. Furthermore, the COVID-19 pandemic offers a natural experiment in those adults who likely had milder AA but did not present to healthcare. Tankel *et al*. have suggested that the reduction which they observed in number of patients admitted with AA during the COVID-19 pandemic could represent successful resolution of mild appendicitis treated symptomatically by patients at home [24]. However safely identifying any such subset of patients requires further research.

The widened paediatric catchment area resulting from a change in referral practice during the pandemic confounds interpretation of the pathology data with respect to paediatric specimens. Noting the contrasting pattern to that evident in adults, this is the most likely explanation for the relative and absolute increase in perforated AA in children, when assessed over the first year of the COVID-19 pandemic. Delayed presentation and resultant delayed diagnosis may also have been contributory, as previously reported by other studies [19,20]. It is also possible that the increased application of a NOM pathway for paediatric patients in the early months of the pandemic may have resulted in a true increase in incidence of perforated AA measured over the 12 month period. This is suggested by a recent meta-analysis of thirteen studies, which reported a significantly higher incidence of complicated appendicitis in children during the COVID-19 pandemic than in the pre-COVID-19 period, accompanying significantly greater use of NOM [22]. However the current study was not designed to address NOM and any proposed link between NOM and subsequent increased risk of complicated appendicitis requires further research.

Another limitation of this study relates to the ability to directly compare with other studies who have adopted a clinical definition and classification of AA. As study cases were derived from those patients who underwent surgery for a presumed diagnosis of AA, generating a pathology specimen, our study findings cannot be generalised to patients clinically diagnosed with AA and who were treated conservatively and did not undergo surgery. Additionally, given that no differences in patient demographics were observed between the two study timeframes evaluated, we did not conduct regression analyses adjusting for these potential confounders. We acknowledge the possibility of residual confounding from unmeasured factors that may explain differences in presentation of patients between the pre- and post-COVID-19 pandemic timeframe. The retrospective nature of data collection from electronic health records may have also been influenced by reporting biases, although it is notable that data completeness was 100% for many variables.

This study has several strengths, including its population-based coverage, although we cannot be sure that the 38% of the Northern Ireland population covered by this study is representative of the entire population. This represents a possible geographical bias that could limit the generalisability of our findings to other settings. Another strength is our robust diagnostic classification, which is based on histopathological assessment, rather than a clinical assessment of complicated versus uncomplicated AA. Again this may have introduced selection bias to our study population, such as the potential for exclusion of milder cases of AA. The ability to compare a full 12 month timeframe helps to avoid seasonal bias, given the long described seasonality of AA incidence [37,38].

In conclusion, the COVID-19 pandemic has challenged the medical profession and continues to do so. With respect to AA, altered public behaviour with respect to seeking healthcare and enforced changes to clinical practice in hospitals have generated interesting data with respect to many aspects of this disease, including its presentation, management and associated pathology. Year 1 of the COVID-19 pandemic was associated with delayed presentation of acute appendicitis in adults and children. In adults, an overall reduction in appendicectomy operations, increased use of pre-operative diagnostic imaging, and fewer specimens showing simple acute appendicitis or non-diagnostic features, collectively support appropriate restriction of surgery for those patients with a more certain acute appendicitis diagnosis. This guidance may be particularly relevant in the event of any future periods of restriction of surgical services.
